# Phylogeographic analysis of *Pseudogymnoascus destructans* partitivirus-pa explains the spread dynamics of white-nose syndrome in North America

**DOI:** 10.1371/journal.ppat.1009236

**Published:** 2021-03-17

**Authors:** Vaskar Thapa, Gregory G. Turner, Marilyn J. Roossinck

**Affiliations:** 1 Department of Plant Pathology and Environmental Microbiology, Center for Infectious Disease Dynamics, Pennsylvania State University, University Park, Pennsylvania, United States of America; 2 Pennsylvania Game Commission, Harrisburg, Pennsylvania, United States of America; Broad Institute of MIT and Harvard, UNITED STATES

## Abstract

Understanding the dynamics of white-nose syndrome spread in time and space is an important component for the disease epidemiology and control. We reported earlier that a novel partitivirus, *Pseudogymnoascus destructans* partitivirus-pa, had infected the North American isolates of *Pseudogymnoascus destructans*, the fungal pathogen that causes white-nose syndrome in bats. We showed that the diversity of the viral coat protein sequences is correlated to their geographical origin. Here we hypothesize that the geographical adaptation of the virus could be used as a proxy to characterize the spread of white-nose syndrome. We used over 100 virus isolates from diverse locations in North America and applied the phylogeographic analysis tool BEAST to characterize the spread of the disease. The strict clock phylogeographic analysis under the coalescent model in BEAST showed a patchy spread pattern of white-nose syndrome driven from a few source locations including Connecticut, New York, West Virginia, and Kentucky. The source states had significant support in the maximum clade credibility tree and Bayesian stochastic search variable selection analysis. Although the geographic origin of the virus is not definite, it is likely the virus infected the fungus prior to the spread of white-nose syndrome in North America. We also inferred from the BEAST analysis that the recent long-distance spread of the fungus to Washington had its root in Kentucky, likely from the Mammoth cave area and most probably mediated by a human. The time to the most recent common ancestor of the virus is estimated somewhere between the late 1990s to early 2000s. We found the mean substitution rate of 2 X 10^−3^ substitutions per site per year for the virus which is higher than expected given the persistent lifestyle of the virus, and the stamping-machine mode of replication. Our approach of using the virus as a proxy to understand the spread of white-nose syndrome could be an important tool for the study and management of other infectious diseases.

## Author summary

White-nose syndrome is a deadly fungal disease of North American bats, that has been spreading from the northeastern United States to Canada and to the south and west. Understanding the spread has been difficult because the fungus is nearly identical throughout North America, consistent with a recent introduction. The fungus is infected with an RNA virus that shows significant variation over time, especially in the gene for the coat protein. Here we used the variation in the virus to create a phylogenetic tree that could help us understand the spread of the fungal disease. We found that the fungus/virus combination was likely in North America since around 1999, earlier than previously thought. In addition, we found that the initial site of introduction was more likely Connecticut, rather than New York State, and that the recently identified fungal infections in bats in Washington state most likely came from Kentucky. This study also demonstrates that viruses of a pathogen can be used to understand the epidemiology of the pathogen.

## Introduction

White-nose syndrome (WNS), caused by the filamentous fungus *Psedogymnoascus destructans* (Pd), is a highly pathogenic infection spreading among North American bats since around 2006 [1–4]. In the last fourteen years, the disease spread over a wide geographic range across the United States with largest movements in southward and westward directions from New York State, where it was first reported [5]. The fungus transmits to healthy bats by contact with infected bats or from the cave environment where the fungus can survive [6,7]. The ability of the fungus to survive for a considerable time without bat hosts [7–10] and at elevated temperatures [8] make the fungus a potential candidate for long-distance movement, most likely by humans through contaminated personal equipment or clothing [3]. The efficiency of transmission may also be impacted by other biological, environmental, and climatic factors. Understanding these variables that drive the spread is important for the epidemiology of WNS and planning conservation efforts to protect bats from WNS. Several models were published recently to explain the spread of WNS [11–14]. However, the models were based on theoretical assumptions and did not capture the dynamic characteristics of the spread over time. Phylogeographic analysis based on phylogenetic relationships of a pathogen over time and space is a valuable tool to characterize the dynamics of spread [15]. This approach not only determines epidemiologically critical dispersal patterns while spreading across the landscape, but also provides evolutionary information.

The North American population of Pd is essentially clonal, represented by a single mating type [16–18]. Studies on single nucleotide polymorphism across the genome and microsatellite data showed some genetic variations in Pd population however, the variations are insufficient to determine the spatial spread of the fungus [19,20]. We reported earlier a partitivirus, *Pseudogymnoascus destructans* partitivirus-pa (PdPV-pa), infection in all Pd isolates that we screened from North American bats with WNS [21]. All partitiviruses (family *Partitiviridae*) like other mycoviruses lack an external infectious phase in their life cycle [22–24]. They are transmitted vertically during cell division and sporogenesis within a host, and via hyphal anastomosis (cell fusion) within the same or closely related vegetatively compatible groups [22]. This lifestyle ensures persistent infection cycles in a host and makes mycoviruses suitable proxies to trace the spread of their hosts based on their genetic signatures. This approach provides a significant tool for understanding the spread of WNS in North America beyond variation in the fungus with its shallow genetic variation. We showed previously, using genetic information of the partitivius from 45 isolates of Pd, that the virus is geographically clustered [21]. Here, we examined the concept with a much larger sample set from across the disease fronts to test the hypothesis that the genetic information from the virus could be used to understand the patterns of WNS spread. We utilized variations in the virus sequences infecting Pd in a Bayesian phylodynamic framework to derive a credible approximation of WNS spread over space and time.

## Materials and methods

### Sample collection

We used 139 Pd isolate samples collected from nine bat species across 17 US states and one province (New Brunswick) of Canada in this study (Table 1). Of the 139 isolates, 45 were from our work published in 2016 [21]. The remainder were from a new collection for this study. The new isolates were obtained as generous donations from many individuals affiliated with various institutions involved in WNS research (Table 1). This sample set represents the maximum available samples we were able to obtain. Although not comprehensive, we are confident that the samples capture the genetic diversity of PdPV, as the sample location, time and bat species were random and included isolates from the early reporting states and the disease front where WNS was advancing. The samples from a few early reporting states like New York and Ohio were from 2008 to 2012 and not from later dates. However, the aim of our analyses was to examine the spatial-temporal dynamics across North America rather than at a state level, so the data coverage justifies our purpose. Moreover, samples from some states like Pennsylvania, West Virginia, Indiana and Kentucky include wide range time-series samples. The samples were obtained in various forms including bat wings, bat wing punches, bat wing swabs, spore suspensions of Pd, total nucleic acid extraction of Pd, or Pd cultures (Table 1). Except for the total nucleic acid extracts, we grew the fungus out of the material received by culturing on Sabouraud dextrose agar (SDA) media to obtain a pure culture of the isolate. The culture conditions and methods to obtain the pure cultures were as described previously [21]. When samples were obtained as total nucleic acid, we enriched the samples for dsRNA followed by RT-PCR to amplify the coat protein segment of PdPV-pa using specific primers as described previously [21].

**Table 1:** Description of *Pseudogymnoascus destructans* (Pd) isolates used in this study.

**S.No**	**Isolate ID**	**Location**	**Date collected**	**Host species**	**Pd isolated from/source**	**Affiliation/Collector or Contact person**
1	1_IN_IB_2012	IN, USA	2012	*Myotis sodalis*	Swab collection	Pennsylvania Game Commission/Greg Turner
2	LB01IN_IN_LB_2015^1^	Wyandotte Cave, Leavenworth, IN	4/20/2015	*Myotis lucifugus*	Bat wing swab	Pennsylvania Game Commission/Greg Turner
3	4WNS_WV_LB_2010	Pocahontas Co, WV, USA	2/23/2010	*Myotis lucifugus*	Spore suspension	University of Georgia/Heather Fenton
4	15WNS_WV_LB_2011	Tucker Co, WV, USA	3/23/2011	*Myotis lucifugus*	Spore suspension	University of Georgia/Heather Fenton
5	20WNS_WV_LB_2011	Hellhole Cave, Pendleton Co, WV, USA	3/23/2011	*Myotis lucifugus*	Spore suspension	University of Georgia/Heather Fenton
6	22WNS_WV_TC_2011	Blackwater Cave, Tucker Co, WV, USA	2/8/2011	*Perimyotis subflavus*	Spore suspension	University of Georgia/Heather Fenton
7	7WNS_WV_LB_2010	Pendleton Co, WV, USA	3/10/2010	*Myotis lucifugus*	Spore suspension	University of Georgia/Heather Fenton
8	8WNS_WV_TC_2010	Greenbrier Co, WV, USA	3/12/2010	*Perimyotis subflavus*	Spore suspension	University of Georgia/Heather Fenton
9	23WNS_WV_LB_2011	Bowden Cave, Randolph Co, USA	3/23/2011	*Myotis lucifugus*	Spore suspension	University of Georgia/Heather Fenton
10	WV1_WV_LB_2010	Hellhole Cave, Pendleton Co, WV, USA	2010	*Myotis lucifugus*	Bat wing pieces	Pennsylvania Game Commission/Greg Turner
11	5WNS_WV_LB_2010	Pocahontas Co, WV, USA	2/23/2010	*Myotis lucifugus*	Spore suspension	University of Georgia/Heather Fenton
12	12WNS_WV_LB_2010	Pendleton Co, WV, USA	10/30/2010	*Myotis lucifugus*	Spore suspension	University of Georgia/Heather Fenton
13	26_WV_TC_2018	New Trout, WV, USA	3/3/2018	*Perimyotis subflavus*	Swab collection	West Virginia Department of Natural Resources/Kieran O’Marley
14	27_WV_TC_2018	New Trout, WV, USA	3/3/2018	*Perimyotis subflavus*	Swab collection	West Virginia Department of Natural Resources/Kieran O’Marley
15	10_IN_TC_2018	Sullivan Cave, Lawrence Co, IN, USA	2/10/2018	*Perimyotis subflavus*	Swab collection	IDNR, Division of Fish & Wildlife/Tim Shier
16	11_IN_LB_2018	Sullivan Cave, Lawrence Co, IN, USA	2/10/2018	*Myotis lucifugus*	Swab collection	IDNR, Division of Fish & Wildlife/Tim Shier
17	20_IN_TC_2018	Buckner Cave, Monroe Co, IN, USA	2/10/2018	*Perimyotis subflavus*	Swab collection	IDNR, Division of Fish & Wildlife/Tim Shier
18	23_IN_TC_2018	Saltpetre Cave, Crawford Co, IN, USA	2/15/2018	*Perimyotis subflavus*	Swab collection	IDNR, Division of Fish & Wildlife/Tim Shier
19	21_IN_TC_2018	Sullivan Cave, Lawrence Co, IN, USA	2/10/2018	*Perimyotis subflavus*	Swab collection	IDNR, Division of Fish & Wildlife/Tim Shier
20	22_IN_LB-2018	Sullivan Cave, Lawrence Co, IN, USA	2/10/2018	*Myotis lucifugus*	Swab collection	IDNR, Division of Fish & Wildlife/Tim Shier
21	25_IN_LB_2018	Sullivan Cave, Lawrence Co, IN, USA	2/10/2018	*Myotis lucifugus*	Swab collection	IDNR, Division of Fish & Wildlife/Tim Shier
22	22954_CT_LB_2010	CT, USA	3/10/2010	*Myotis lucifugus*	Total nucleic acid	National Wildlife Health Center/Jeffery M. Lorch
23	TC3_PA_TC_2014	Sabula RR Tunnel, PA, USA	1/1/2014	*Perimyotis subflavus*	Bat wing pieces	Pennsylvania Game Commission/Greg Turner
24	TC4_PA_TC_2014	Sabula RR Tunnel, PA, USA	1/1/2014	*Perimyotis subflavus*	Bat wing pieces	Pennsylvania Game Commission/Greg Turner
25	15_VA_TC_2018	Spanglers Cave, Lee Co, VA, USA	1/30/2018	*Perimyotis subflavus*	Swab collection	Virginia Department of Game and Inland Fisheries/Rick Reynolds
26	22467_VA_LB_2009	VA, USA	3/4/2009	*Myotis lucifugus*	Total nucleic acid	National Wildlife Health Center/Jeffery M. Lorch
27	2_WA_YM_2017	Rattlesnake Lake, King Co, WA, USA	8/4/2017	*Myotis thysanodes*	Fungal culture	University of California, Davis/Piyaporn Eiamcharoen
28	21_WA_YM_2018	Rattlesnake Lake, King Co, WA, USA	6/11/2018	*Myotis yumanensis/lucifugus*	Spore suspension	University of California, Davis/Piyaporn Eiamcharoen
29	40_WA_YM_2018	Valley Camp, King Co, WA, USA	8/14/2018	*Myotis yumanensis/lucifugus*	Spore suspension	University of California, Davis/Piyaporn Eiamcharoen
30	29_WA_YM_2018	Cedar River Education Center, King Co, WA, USA	7/10/2018	*Myotis yumanensis/lucifugus*	Spore suspension	University of California, Davis/Piyaporn Eiamcharoen
31	32_WA_YM_2018	Tinkham bridge, King Co, WA, USA	7/10/2018	*Myotis yumanensis/lucifugus*	Spore suspension	University of California, Davis/Piyaporn Eiamcharoen
32	37_WA_YM_2018	Cedar River Education Center, King Co, WA, USA	7/27/2018	*Myotis yumanensis*	Spore suspension	University of California, Davis/Piyaporn Eiamcharoen
33	27099_WA_LB_2016	King Co, WA, USA	3/11/2016	*Myotis lucifugus*	Total nucleic acid	National Wildlife Health Center/Jeffery M. Lorch
34	27WNS_KY_NLE_2012	B&O Cave, Breckinridge Co, KY, USA	1/13/2012	*Myotis septentrionalis*	Spore suspension	University of Georgia/Heather Fenton
35	34_WA_YM_2018	Cedar River Education Center, King Co, WA, USA	7/10/2018	*Myotis yumanensis*	Spore suspension	University of California, Davis/Piyaporn Eiamcharoen
36	5_KY_TC_2018	Misty Cave< Jackson Co, KY, USA	1/25/2018	*Perimyotis subflavus*	Swab collection	Department of Fish and Wildlife Resources/Sunni Carr
37	16_KY_LB_2018	Smokehole Cave, Rockcastle Co, KY, USA	1/29/2018	*Myotis lucifugus*	Swab collection	Department of Fish and Wildlife Resources/Sunni Carr
38	17_KY_TC_2018	Barefoot Saltpeter Cave, Wayne Co, KY, USA	1/22/2018	*Perimyotis subflavus*	Swab collection	Department of Fish and Wildlife Resources/Sunni Carr
**S.No**	**Isolate ID**	**Location**	**Date collected**	**Host species**	**Pd isolated from/source**	**Affiliation/Collector or Contact person**
39	19_KY_LB_2018	Smokehole Cave, Rockcastle Co, KY, USA	1/29/2018	*Myotis lucifugus*	Swab collection	Department of Fish and Wildlife Resources/Sunni Carr
40	26WNS_KY_TC_2012	Norton Valley Cave, Breckinridge Co, KY, USA	1/19/2012	*Perimyotis subflavus*	Spore suspension	University of Georgia/Heather Fenton
41	32WNS_KY_TC_2012	Triple S Cave, Wayne Co, KY, USA	2/14/2012	*Perimyotis subflavus*	Spore suspension	University of Georgia/Heather Fenton
42	31WNS2_KY_TC_2012	Penitentiary Cave, Breckinridge Co, KY, USA	1/23/2012	*Perimyotis subflavus*	Spore suspension	University of Georgia/Heather Fenton
43	36WNS_KY_TC_2012	Peter Cave, Wayne Co, KY, USA	2/16/2012	*Perimyotis subflavus*	Spore suspension	University of Georgia/Heather Fenton
44	61WNS_KY_NLE_2013	Colossal Cave, Edmonson Co, KY, USA	1/8/2013	*Myotis septentrionalis*	Spore suspension	University of Georgia/Heather Fenton
45	21ST96_KY_TC_2011	Cool Springs Cave, Trigg Co, KY, USA	4/19/2011	*Perimyotis subflavus*	Spore suspension	University of Georgia/Heather Fenton
46	30_WA_YM_2018	Cedar River Education Center, King Co, WA, USA	7/27/2018	*Myotis yumanensis*	Spore suspension	University of California, Davis/Piyaporn Eiamcharoen
47	41-WA_YM_2018	Maple Valley, King Co, WA, USA	8/14/2018	*Myotis yumanensis/lucifugus*	Spore suspension	University of California, Davis/Piyaporn Eiamcharoen
48	35WNS_KY_LB_2012	Buzzard Cave, Breckinridge Co, KY, USA	1/19/2012	*Myotis lucifugus*	Spore suspension	University of Georgia/Heather Fenton
49	2SE092_PA_LB_2009	Dunmore Slope Co, PA, USA	2009	*Myotis lucifugus*	Spore suspension	University of Georgia/Heather Fenton
50	BB06_PA_BB_2015^1^	Layton Fire Clay Mine, Allegheny Co, PA	3/4/2015	*Eptesicus fuscus*	Bat wing punch	Pennsylvania Game Commission/Greg Turner
51	BB10_PA_BB_2015^1^	Layton Fire Clay Mine, Allegheny Co, PA	3/4/2015	*Eptesicus fuscus*	Bat wing punch	Pennsylvania Game Commission/Greg Turner
52	LB01_PA_LB_2011^1^	Blossburg Mine, Tioga Co, PA	3/22/2011	*Myotis lucifugus*	Bat wing pieces	Pennsylvania Game Commission/Greg Turner
53	LB05_PA_LB_2012^1^	Centre Co, PA	3/28/2012	*Myotis lucifugus*	Bat wing pieces	Pennsylvania Game Commission/Greg Turner
54	LB04_PA_LB_2012^1^	Centre Co, PA	3/25/2012	*Myotis lucifugus*	Bat wing pieces	Pennsylvania Game Commission/Greg Turner
55	LB07_PA_LB_2012^1^	Cook Forest State Park, Cooksburg, PA	3/21/2012	*Myotis lucifugus*	Bat wing pieces	Pennsylvania Game Commission/Greg Turner
56	LB08_PA_LB_2012^1^	Cook Forest State Park, Cooksburg, PA	3/21/2012	*Myotis lucifugus*	Bat wing pieces	Pennsylvania Game Commission/Greg Turner
57	TC01_PA_TC_2011^1^	Blossburg Mine, Tioga Co, PA	3/22/2011	*Perimyotis subflavus*	Bat wing pieces	Pennsylvania Game Commission/Greg Turner
58	LB03_PA_LB_2013^1^	Indian Cave, Somerset Co, PA	2/16/2013	*Myotis lucifugus*	Bat wing pieces	Pennsylvania Game Commission/Greg Turner
59	LB02_PA_LB_2012^1^	Kennerdell, PA	3/13/2012	*Myotis lucifugus*	Bat wing pieces	Pennsylvania Game Commission/Greg Turner
60	LBB_PA_LB_2011^1^	Blossburg Mine, Tioga Co, PA	3/22/2011	*Myotis lucifugus*	Bat wing pieces	Pennsylvania Game Commission/Greg Turner
61	LB55617_PA_LB_2014^1^	Canoe Creek, Holidaysburg, PA	4/24/2014	*Myotis lucifugus*	Bat wing swab	Pennsylvania Game Commission/Greg Turner
62	LB55571_PA_LB_2014^1^	Canoe Creek, Holidaysburg, PA	4/9/2014	*Myotis lucifugus*	Bat wing swab	Pennsylvania Game Commission/Greg Turner
63	TC2_PA_TC_2014	Sabula RR Tunnel, PA, USA	1/1/2014	*Perimyotis subflavus*	Bat wing pieces	Pennsylvania Game Commission/Greg Turner
64	LB06_PA_LB_2012^1^	Cook Forest State Park, Cooksburg, PA	3/21/2012	*Myotis lucifugus*	Bat wing pieces	Pennsylvania Game Commission/Greg Turner
65	2063121_NY_LB_2008^1^	Williams Hotel, NY	2008	*Myotis lucifugus*	Fungal culture	Northern Research Center/Daniel Lindner
66	M2461_NY_LB_2010^1^	NY	5/11/2010	*Myotis lucifugus*	Fungal culture	Northern Research Center/Daniel Lindner
67	M2332_NY_LB_2009^1^	Dannemora, Clinton, NY	3/11/2009	*Myotis lucifugus*	Fungal culture	Northern Research Center/Daniel Lindner
68	M2334_NY_LB_2009^1^	Newstead, Erie, NY	3/12/2009	*Myotis lucifugus*	Fungal culture	Northern Research Center/Daniel Lindner
69	M2333_NY_LB_2009^1^	Dannemora, Clinton, NY	3/11/2009	*Myotis lucifugus*	Fungal culture	Northern Research Center/Daniel Lindner
70	M3902_WV_LB_2010^1^	WV	2/23/2010	*Myotis lucifugus*	Fungal culture	Northern Research Center/Daniel Lindner
71	M3903_WV_TC_2010^1^	WV	3/12/2010	*Perimyotis subflavus*	Fungal culture	Northern Research Center/Daniel Lindner
72	M3911_WV_TC_2011^1^	WV	3/11/2011	*Perimyotis subflavus*	Fungal culture	Northern Research Center/Daniel Lindner
73	M3910_WV_LB_2011^1^	WV	3/23/2011	*Myotis lucifugus*	Fungal culture	Northern Research Center/Daniel Lindner
74	M3912_WV_LB_2011^1^	WV	3/23/2011	*Myotis lucifugus*	Fungal culture	Northern Research Center/Daniel Lindner
75	M2335_NY_LB_2009^1^	Ithaca, Tompkins, NY	3/16/2009	*Myotis lucifugus*	Fungal culture	Northern Research Center/Daniel Lindner
76	14WNS_NC_TC_2011	Cranberry Iron Mine, Avery Co, NC, USA	2/3/2011	*Perimyotis subflavus*	Spore suspension	University of Georgia/Heather Fenton
77	24WNS_NC_TC_2011	Pseudosaltpeter Cave, McDowell, NC, USA	4/5/2011	*Perimyotis subflavus*	Spore suspension	University of Georgia/Heather Fenton
78	16WNS_NC_LB_2011	Isom Iron Mine, Yancey Co, NC, USA	2/8/2011	*Myotis lucifugus*	Spore suspension	University of Georgia/Heather Fenton
79	30WNS_NC_TC_2012	Big Ridge Mine, Haywood Co, NC, USA	2/9/2012	*Perimyotis subflavus*	Spore suspension	University of Georgia/Heather Fenton
**S.No**	**Isolate ID**	**Location**	**Date collected**	**Host species**	**Pd isolated from/source**	**Affiliation/Collector or Contact person**
80	M3905_NC_LB_2011^1^	NC	2/3/2011	*Myotis lucifugus*	Fungal culture	Northern Research Center/Daniel Lindner
81	M3908_NC_LB_2011^1^	NC	2/8/2011	*Myotis lucifugus*	Fungal culture	Northern Research Center/Daniel Lindner
82	M3906_NC_TC_2011^1^	NC	2/3/2011	*Perimyotis subflavus*	Fungal culture	Northern Research Center/Daniel Lindner
83	1SE091_WV_LB_2009	Pendleton Co, WV, USA	2009	*Myotis lucifugus*	Spore suspension	University of Georgia/Heather Fenton
84	M3907_WV_LB_2011^1^	WV	3/23/2011	*Myotis lucifugus*	Fungal culture	Northern Research Center/Daniel Lindner
85	M4513_VT_LB_2015^1^	VT	-	*Myotis lucifugus*	Fungal culture	Northern Research Center/Daniel Lindner
86	NLE01VT_VT_NLE_2015^1^	Plymouth Cave, Plymouth, VT	3/26/2015	*Myotis septentrionalis*	Bat wing pieces	Pennsylvania Game Commission/Greg Turner
87	M4514_VT_LB_2015^1^	VT	-	*Myotis lucifugus*	Fungal culture	Northern Research Center/Daniel Lindner
88	24290_MO_LB_2013	MO, USA	2/26/2013	*Myotis lucifugus*	Total nucleic acid	National Wildlife Health Center/Jeffery M. Lorch
89	44WNS_TN_TC_2012	Bellamy Cave, Montgomery Co, TN, USA	3/1/2012	*Perimyotis subflavus*	Spore suspension	University of Georgia/Heather Fenton
90	53WNS_TN_TC_2012	Carlton Cave, Franklin Co, TN, USA	3/2/2012	*Perimyotis subflavus*	Spore suspension	University of Georgia/Heather Fenton
91	54WNS_TN_TC_2012	Battlefield Pit #1 Cave, Hamilton Co, TN, USA	4/17/2012	*Perimyotis subflavus*	Spore suspension	University of Georgia/Heather Fenton
92	70WNS_TN_TC_2013	Clairbome Co, TN, USA	3/15/2013	*Perimyotis subflavus*	Spore suspension	University of Georgia/Heather Fenton
93	45WNS_TN_TC_2012	Carlton Cave, Franklin Co, TN, USA	3/2/2012	*Perimyotis subflavus*	Spore suspension	University of Georgia/Heather Fenton
94	51WNS_TN_GB_2012	Pearson Cave, Hawkins Co, TN, USA	3/29/2012	*Myotis grisescens*	Spore suspension	University of Georgia/Heather Fenton
95	47WNS_TN_TC_2012	White Oak Blowhole Cave, Blount Co, TN, USA	3/14/2012	*Perimyotis subflavus*	Spore suspension	University of Georgia/Heather Fenton
96	52WNS_TN_TC_2012	Bellamy Cave, Montgomery Co, TN, USA	3/1/2012	*Perimyotis subflavus*	Spore suspension	University of Georgia/Heather Fenton
97	46WNS_TN_LB_2012	White Oak Blowhole Cave, Blount Co, TN, USA	3/14/2012	*Myotis lucifugus*	Spore suspension	University of Georgia/Heather Fenton
98	64WNS_TN_LB_2013	Houston Co, TN, USA	1/18/2013	*Myotis lucifugus*	Spore suspension	University of Georgia/Heather Fenton
99	49WNS_TN_TC_2012	Upstream Cave, Hancock Co, TN, USA	3/29/2012	*Perimyotis subflavus*	Spore suspension	University of Georgia/Heather Fenton
100	69WNS_TN_LB_2013	Campbell Co, TN, USA	4/15/2013	*Myotis lucifugus*	Spore suspension	University of Georgia/Heather Fenton
101	67WNS_TN_LB_2013	Fentress Co, TN, USA	1/23/2013	*Myotis lucifugus*	Spore suspension	University of Georgia/Heather Fenton
102	23444_TN_LB_2011	TN, USA	2/11/2011	*Myotis lucifugus*	Total nucleic acid	National Wildlife Health Center/Jeffery M. Lorch
103	71WNS_SC_SFB_2013	Pickens Co, SC, USA	4/2/2013	*Myotis leibii*	Spore suspension	University of Georgia/Heather Fenton
104	44767_KY_LB_2014	KY, USA	3/14/2014	*Myotis lucifugus*	Total nucleic acid	National Wildlife Health Center/Jeffery M. Lorch
105	21201_Can_LB_2012^1^	White Cave, New Brunswick, Canada	2012	*Myotis lucifugus*	Fungal culture	New Brunswick Museum/Karen J. Vanderwolf
106	82205_Can_LB_2012^1^	White Cave, New Brunswick, Canada	2012	*Myotis lucifugus*	Fungal culture	New Brunswick Museum/Karen J. Vanderwolf
107	92203_Can_LB_2012^1^	White Cave, New Brunswick, Canada	2012	*Myotis lucifugus*	Fungal culture	New Brunswick Museum/Karen J. Vanderwolf
108	212104_Can_NLE_2012^1^	Markhamville Mine, New Brunswick, Canada	2012	*Myotis septentrionalis*	Fungal culture	New Brunswick Museum/Karen J. Vanderwolf
109	702107_Can_TC_2013^1^	Markhamville Mine, New Brunswick, Canada	2013	*Perimyotis subflavus*	Fungal culture	New Brunswick Museum/Karen J. Vanderwolf
110	421101_Can_NLE_2012^1^	Harbell Cave, New Brunswick, Canada	2012	*Myotis septentrionalis*	Fungal culture	New Brunswick Museum/Karen J. Vanderwolf
111	461202_Can_TC_2012^1^	Glebe Mine, New Brunswick, Canada	2012	*Perimyotis subflavus*	Fungal culture	New Brunswick Museum/Karen J. Vanderwolf
112	681102_Can_TC_2013^1^	Glebe Mine, New Brunswick, Canada	2013	*Perimyotis subflavus*	Fungal culture	New Brunswick Museum/Karen J. Vanderwolf
113	642103_Can_LB_2012^1^	Berryton Cave, New Brunswick, Canada	2012	*Myotis lucifugus*	Fungal culture	New Brunswick Museum/Karen J. Vanderwolf
114	671105_Can_TC_2013^1^	Glebe Mine, New Brunswick, Canada	2013	*Perimyotis subflavus*	Fungal culture	New Brunswick Museum/Karen J. Vanderwolf
115	1-PA_TC_2018	Casparis Mine, Fayette Co, PA, USA	2/6/2018	*Perimyotis subflavus*	Swab collection	Pennsylvania Game Commission/Greg Turner
116	15_PA_TC_2018	Casparis Mine, Fayette Co, PA, USA	2/6/2018	*Perimyotis subflavus*	Swab collection	Pennsylvania Game Commission/Greg Turner
117	17_PA_LB_2018	Alexander Cave, Mifflin Co, PA, USA	2/26/2018	*Myotis lucifugus*	Swab collection	Pennsylvania Game Commission/Greg Turner
118	17WNS_OH_LB_2011	Woody Mine, Lawrence Co, OH, USA	3/22/2011	*Myotis lucifugus*	Spore suspension	University of Georgia/Heather Fenton
119	28WNS_OH_LB_2012	Park Cave, Geauga Co, OH, USA	2/1/2012	*Myotis lucifugus*	Spore suspension	University of Georgia/Heather Fenton
120	33WNS_OH_LB_2012	Lewisburg Mine, Preble Co, OH, USA	2/23/2012	*Myotis lucifugus*	Spore suspension	University of Georgia/Heather Fenton
**S.No**	**Isolate ID**	**Location**	**Date collected**	**Host species**	**Pd isolated from/source**	**Affiliation/Collector or Contact person**
121	34WNS_OH_LB_2012	Lewisburg Mine, Preble Co, OH, USA	2/23/2012	*Myotis lucifugus*	Spore suspension	University of Georgia/Heather Fenton
122	37WNS_OH_LB_2012	Park Cave, Cuyahoga Co, OH, USA	2/2/2012	*Myotis lucifugus*	Spore suspension	University of Georgia/Heather Fenton
123	38WNS_OH_LB_2012	House Cave, Portage Co, OH, USA	2/14/2012	*Myotis lucifugus*	Spore suspension	University of Georgia/Heather Fenton
124	M3909_OH_LB_2011^1^	OH, USA	3/22/2011	*Myotis lucifugus*	Fungal culture	Northern Research Center/Daniel Lindner
125	72WNS_GA_TC_2013	Dade Co, GA, USA	3/4/2013	*Perimyotis subflavus*	Spore suspension	University of Georgia/Heather Fenton
126	4_KYmc_TC_2018	JT Cave, KY, USA	2/6/2018	*Perimyotis subflavus*	Swab collection	Mammoth Cave NP/Rickard S. Toomey
127	M2443_NY_TC_2010^1^	NY	4/13/2010	*Perimyotis subflavus*	Fungal culture	Northern Research Center/Daniel Lindner
128	7_KY_TC-2018	Wind Cave, Wayne Co, KY, USA	1/30/2018	*Perimyotis subflavus*	Swab collection	Department of Fish and Wildlife Resources/Sunni Carr
129	29WNS_KY_LB_2012	Roberts Hollow Cave, Breckinridge Co, KY, USA	2/3/2012	*Myotis lucifugus*	Spore suspension	University of Georgia/Heather Fenton
130	65WNS_KY_LB_2013	Edmonson Co, KY, USA	2/12/2013	*Myotis lucifugus*	Spore suspension	University of Georgia/Heather Fenton
131	66WNS_KY_TC_2013	Hart Co, KY, USA	2/13/2013	*Perimyotis subflavus*	Spore suspension	University of Georgia/Heather Fenton
132	63WNS_KY_LB_2013	Letcher Co, KY, USA	1/25/2013	*Myotis lucifugus*	Spore suspension	University of Georgia/Heather Fenton
133	55WNS_KY_LB_2013	Lee Cave, Edmonson Co, KY, USA	2/1/2013	*Myotis lucifugus*	Spore suspension	University of Georgia/Heather Fenton
134	24271_IL_LB_2013	IL, USA	2/7/2013	*Myotis lucifugus*	Total nucleic acid	National Wildlife Health Center/Jeffery M. Lorch
135	58WNS_AL_TC_2013	Fern Cave, Jackson Co, AL, USA	3/21/2013	*Perimyotis subflavus*	Spore suspension	University of Georgia/Heather Fenton
136	59WNS_AL_TC_2013	Fern Cave, Jackson Co, AL, USA	3/21/2013	*Perimyotis subflavus*	Spore suspension	University of Georgia/Heather Fenton
137	44797_AL_TC_2015	AL, USA	2/11/2015	*Perimyotis subflavus*	Total nucleic acid	National Wildlife Health Center/Jeffery M. Lorch
138	18_KYmc_TC_2018	Carmichael, Rocky Mountain, KY, USA	2/1/2018	*Perimyotis subflavus*	Swab collection	Mammoth Cave NP/Rickard S. Toomey
139	2_PA_LB_2018	Casparis Mine, Fayette Co, PA, USA	2/6/2018	*Myotis lucifugus*	Swab collection	Pennsylvania Game Commission/Greg Turner

1 Isolates from our previous study [21].

### Viral RNA amplification, sequence determination and sequence analysis

The dsRNAs of PdPV-pa infecting North American isolates of Pd, were extracted from fungal lyophilized tissue, followed by RT-PCR to amplify the coat protein gene of the virus, and direct sequence analysis, as described previously [21]. The primer pair (5’ ACTCTGTGTTAACGGAGG 3’F, 5’ CTGTAGTTGACACCTGTACC3’ R) amplified 1224 bp of PdPV-pa coat protein; sequences were trimmed to 1088 bp to avoid noise. Nucleotide sequences were aligned using the multiple alignment fast fourier transform (MAFFT) algorithm in Geneious 10.2.6 [25]. We manually edited the sequences whenever necessary, particularly when the frame was shifted, to improve the alignment quality.

### Phylogeographic analysis of PdPV-pa

A phylogeographic analysis using the coat protein sequences of PdPV-pa was performed in BEAST v2.5 [26]. The analysis is based on the Bayesian framework and includes molecular-clock, evolutionary, and spatial models. Before conducting the analysis, the temporal signals of our molecular data were checked in the TempEst tool [27]. A Bayesian non-clock phylogeny was constructed in Geneious 10.2.6 using Hasegawa-Kishino-Yano (HKY) nucleotide substitution model with gamma distribution rates among sites. Then, a correlation between the genetic divergence and the sampling date was plotted in TempEst (S1 Fig) using the consensus tree file generated in Geneious.

After confirming the positive temporal signature of our data, we used a discrete phylogeographic model in BEAST. The discrete phylogeographic model was appropriate because our data were time referenced and spatially descriptive. The sampling dates were entered as years and the sample locations were added as discrete traits. We used the HKY substitution model with a kappa value 2 and gamma category count of 4. HKY (121321, slight variance in r_cg_ rate from HKY) was the most supported model (38% posterior support within 95% highest posterior density, HPD) (S2 Fig) out of 30 reversible models with symmetric rate matrices tested in bModelTest in BEAST [28]. A strict clock model with the prior set to coalescent constant population was used in the analysis. Markov chain Monte Carlo (MCMC) algorithms were run for 250 million states and sampled every 25,000 states. MCMC conversions were evaluated using Tracer v1.7 [29]. The phylogenies with the posterior distribution values were summarized in TreeAnnonator applying maximum clade credibility (MCC) trees excluding 10% burn-in. The consensus tree was visualized in FigTree v1.4.4 [30], using midpoint rooting, based on the assumption of a constant evolutionary rate with the strict clock model [15].

Spatial phylogenetic and reconstruction of evolutionary dynamics (SPREAD) application was used to map spatial diffusion and 95% HPD contours of the virus spread obtained from Bayesian inference [31]. SPREAD version 1.0.7 was used for all visualization in this study. The posterior summaries of diffusion from the MCC tree of BEAST were associated with the discrete locations and exported to a keyhole markup language (KML) file that was viewed in Google Earth [32]. We also performed the Bayesian stochastic search variable selection (BSSVS) analysis and visualized in SPREAD to determine the locations that were significant for PdPV-pa dispersal [33]. In the analysis Bayes factors (BF), indicating deviation between the prior beliefs and the posteriors, distinguished the locations that have higher probabilities than expected [34]. We plotted the location pairs having BF≥3 in Google Earth for visualization. Data was manually transferred to a USGS map for visualization in the figures.

## Results

### Phylogenetic history of PdPV-pa in North America

**Fig 1:**
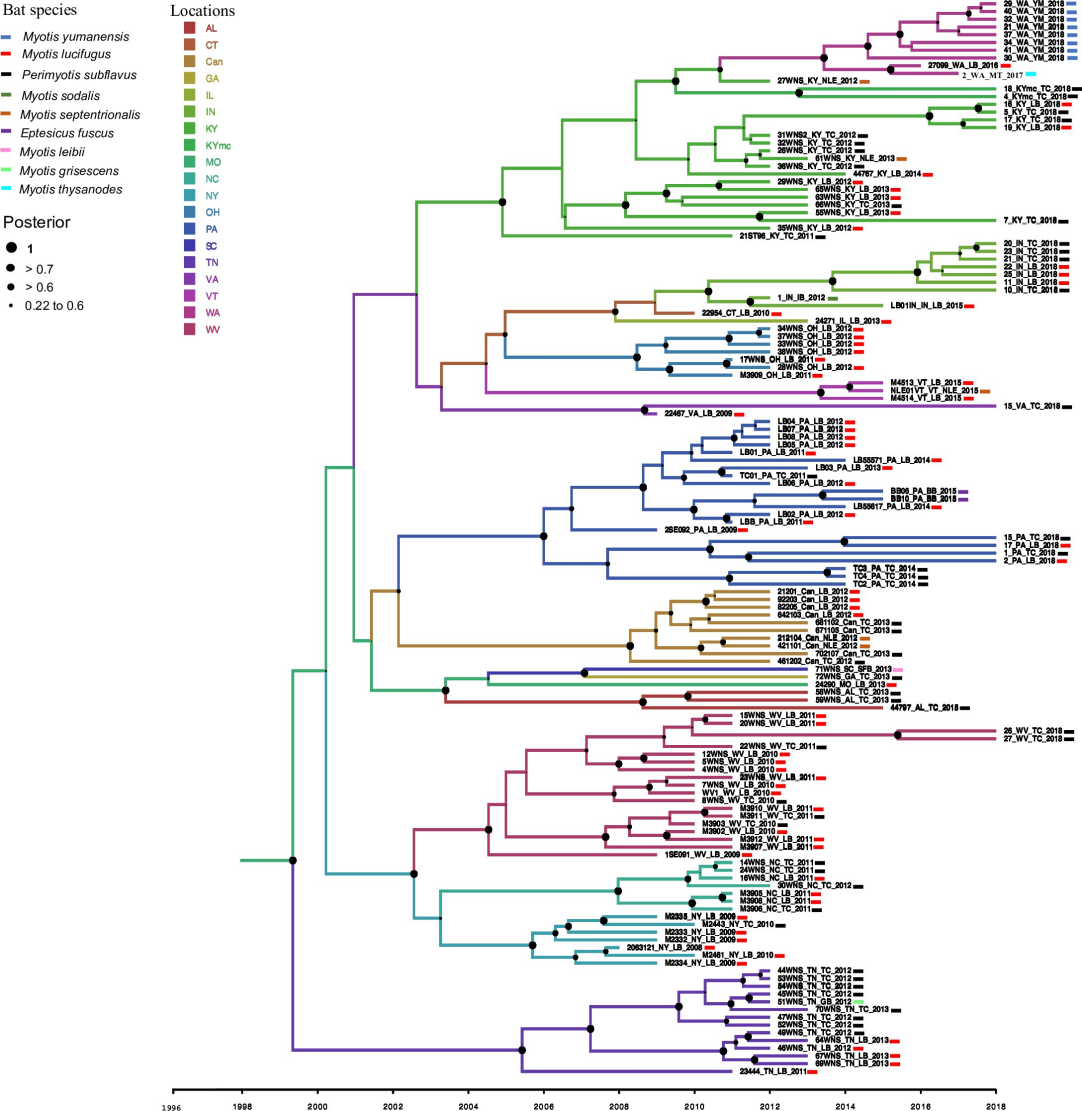
Strict clock phylogeographic tree constructed from the coat protein sequence of 139 PdPV-pa isolates in BEAST v2.5. The tree is annotated with colors coded for the locations that included 17 US states and New Brunswick, Canada. Standard abbreviations of US states are used in the figure legend except “Can” for New Brunswick province of Canada and “KYmc” for Mammoth cave locations in Kentucky. Bat species are notes according to the legend. Nodes of the tree are denoted by solid black circles and the size of the circle corresponds to posterior supports shown in the top legend. Branch lengths are in time, depicted by a horizontal time-scale and the branch thickness corresponds to posterior values. For a detail description of the virus isolate ID, refer to Table 1.

**Fig 2:**
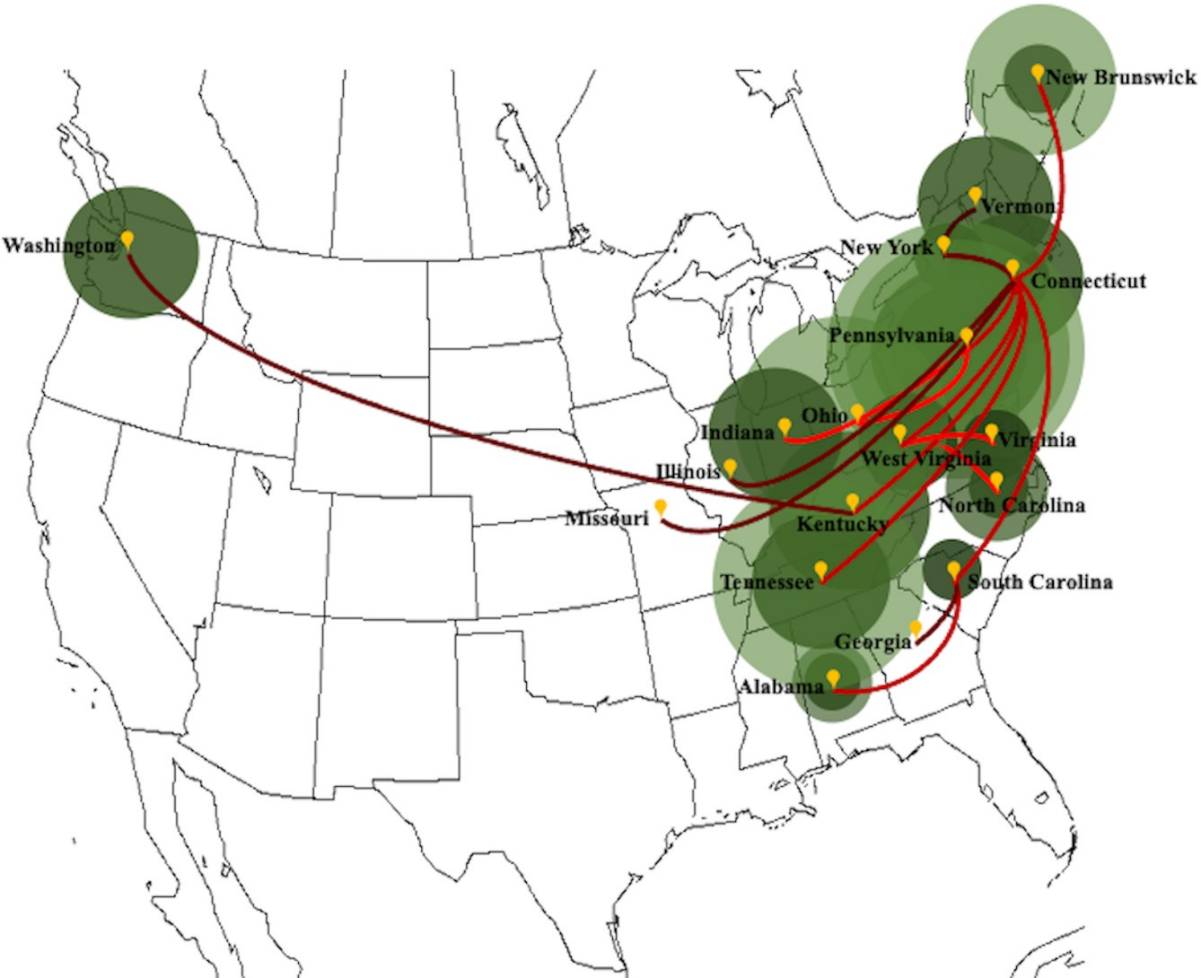
Spread of white-nose syndrome in North America as predicted from PdPV*-*pa phylogeographic analysis in BEAST and visualized in Google Earth with SPREAD v1.0.7. The area under concentric circles represents the number of branches in that particular location at that time shown by different shades of green. The lines connecting different locations represent branches in the maximum clade credibility tree. The bright to dark gradient of the line’s red color represents the time from 1999 to 2018.

**Fig 3:**
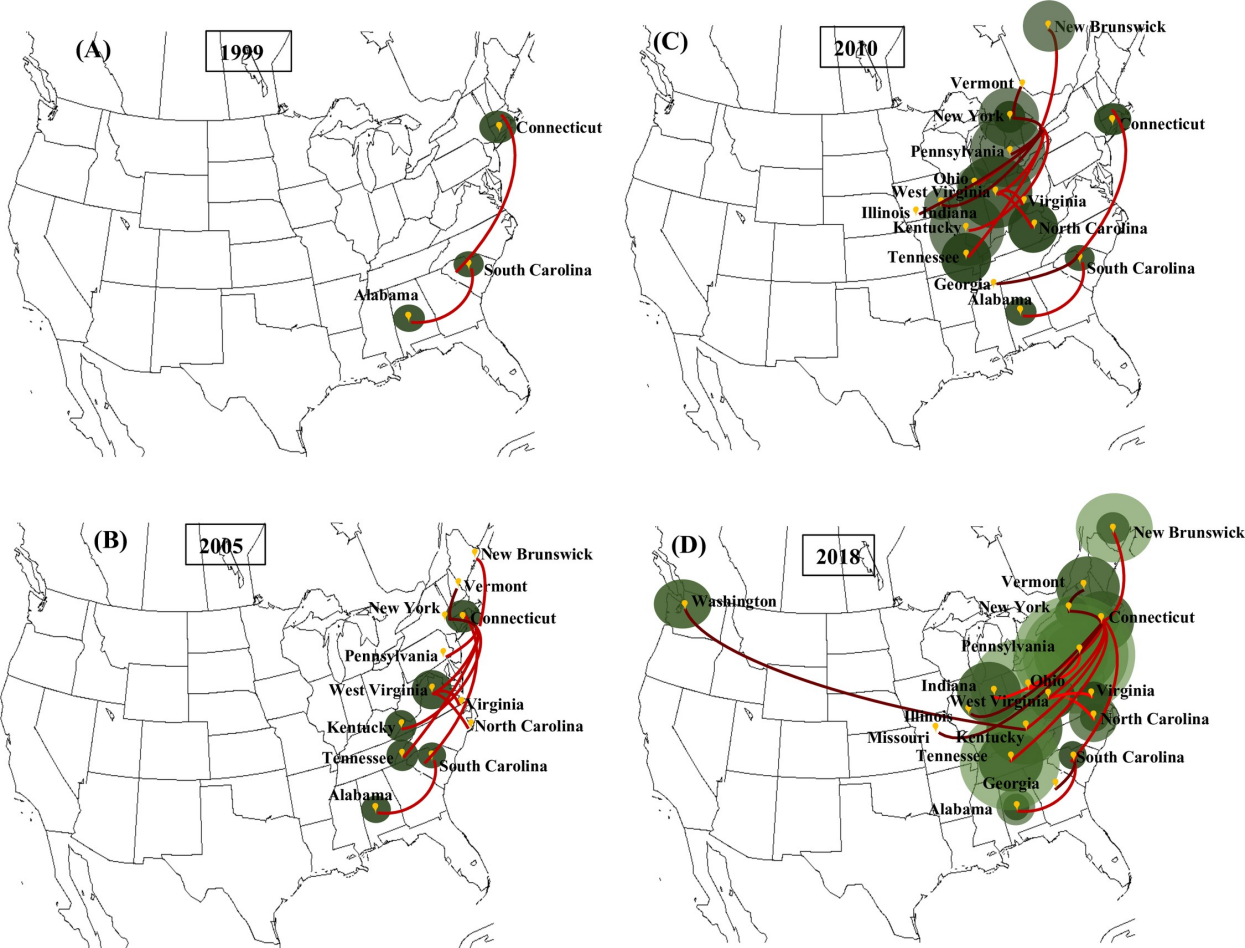
Temporal dynamics of white-nose syndrome spread as inferred from PdPV-pa phylodynamic plotted in Google Earth using SPREAD v1.0.7. Snapshot of (A) spread pattern by 1999, (B) spread pattern by 2005, (C) spread pattern by 2010 and (D) spread pattern by 2018. Lines between the locations represent branch in the maximum clade credibility (MCC) tree. The brightness to the dark color gradient of red indicates the relative age of the dispersal (old to recent).

The strict clock phylogeographic tree constructed with the coat protein sequences from 139 isolates of PdPV-pa in BEAST illustrated geographical clustering (Fig 1). We found well-supported clades (posterior probability, PP ≥ 0.7 to 1) for all US states and New Brunswick in Canada. The virus isolates sampled were associated with nine Pd infecting species of bats (Table 1). Unlike geographical signatures, the virus variation showed no relationships with the bat species (Fig 1), implying that the fungus is transmitting without any genetic barrier among bat species. Some inner nodes of the tree closer to the root had PP values ranging from 0.22 to 0.6 (Fig 1). The clade associated with Tennessee showed strong posterior support with the root node that also appeared as a separate branch from the rest of the clades. In the tree, the branches are annotated with colors corresponding to the respective ancestral locations. No single ancestral location was found dominating across the tree topology. The temporal dynamics of PdPV-pa spread based on the discrete-trait (location) model was visualized with SPREAD v.1.0.7 (Fig 2). The area under concentric circles represents the number of branches in that particular location at that time and the lines connecting different locations represent branches in the MCC tree. Our sample size for the virus isolate corresponds to a particular location and thus matches well with the total area under the circles. However, the areas of the circles before our sampling time (before 2008) are the projected measure of the virus diversity at the ancestral locations. For example, during 1999, the virus appears to have existed in Connecticut, Alabama, and South Carolina (Fig 3A). By 2005, many branches emerged from Connecticut and established the virus in New York, Pennsylvania, West Virginia, Kentucky, and Tennessee (Fig 3B). Virginia and North Carolina were connected from the branches that emerged from West Virginia and Vermont connected from a branch from New York during that period. By 2010, the virus moved to New Brunswick, Ohio, Indiana, Illinois, and Georgia (Fig 3C). By 2018, the virus had traveled to the rest of the sampled states including a long-distance jump from Kentucky to Washington (Fig 3D). The analysis indicates PdPV-pa presence in many sampled states before WNS was first reported in 2006, and Connecticut, West Virginia, New York, and Kentucky appeared as major source locations with multiple connectivity for the virus spread.

### Inference on long-distance PdPV-pa spread

**Fig 4:**
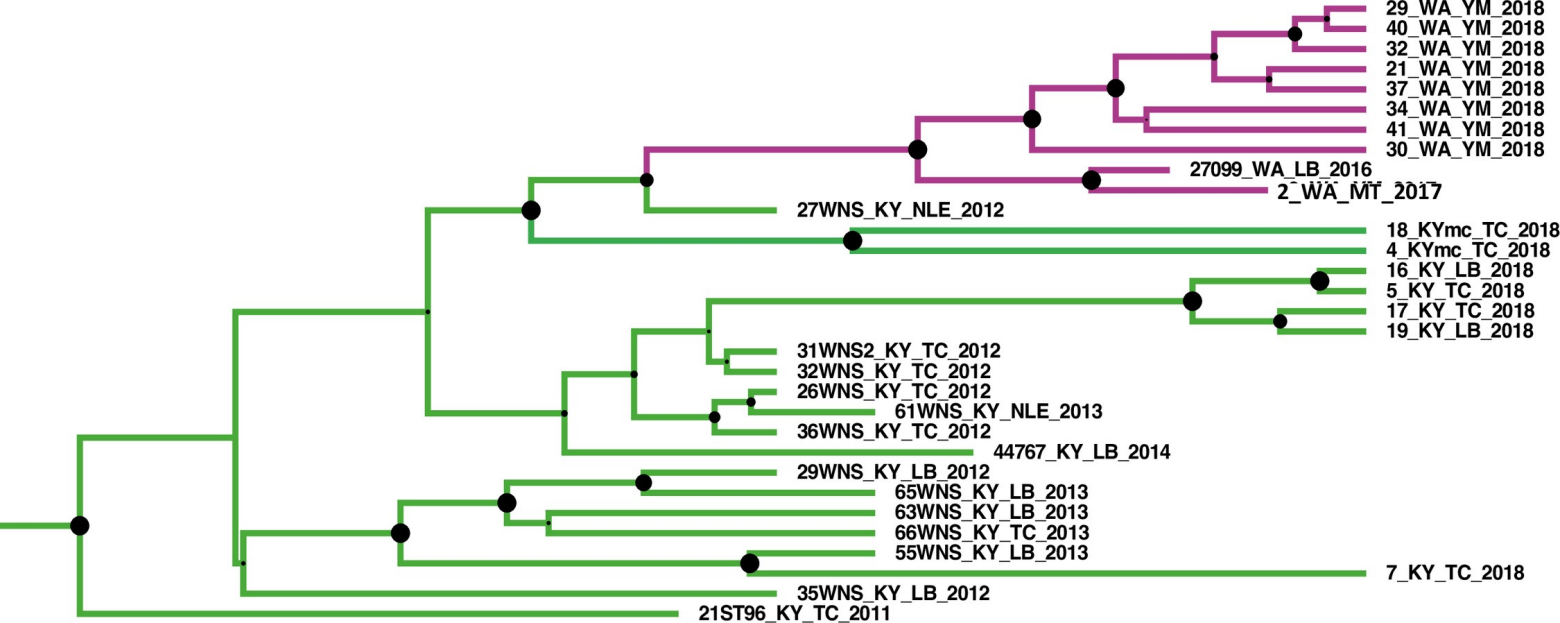
Zoom-in view of the Kentucky-Washington clade from the summary maximum clade credibility (MCC) tree in Fig 1. The 27WNS_KY_NLE_2012 isolate from Kentucky is phylogenetically close to the isolates sampled from Washington. All these isolates along with Mammoth cave isolates (18KYmc_TC_2018 and 4KYmc_TC_2018) shared a common ancestor. The number close to each node corresponds to the posterior support value which is also graphically represented by the size of the solid black circle in each node.

**Fig 5:**
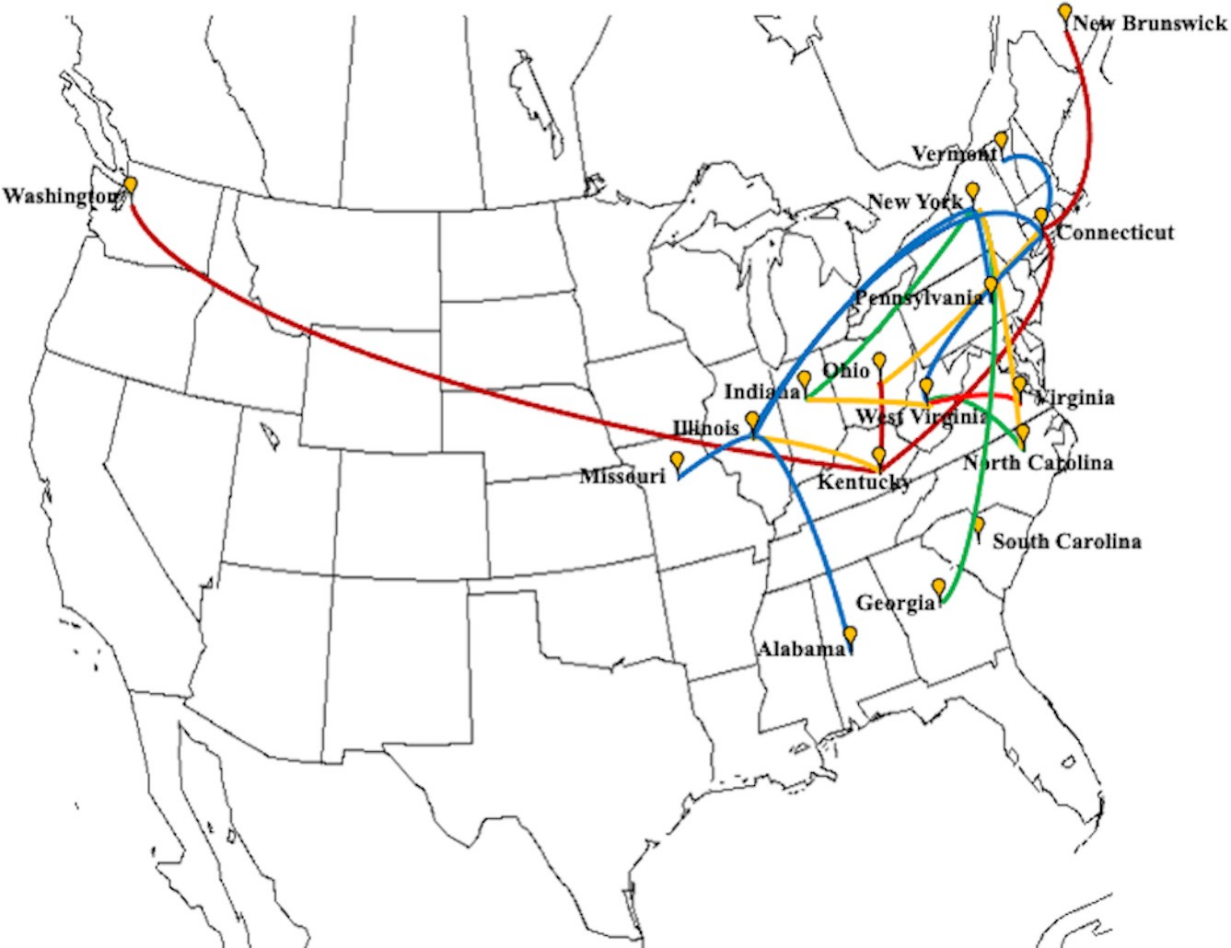
Map showing connections between significant location pairs with BF ≥ 3 as determined by BSSVS analysis. Blue line color corresponds to BF = 3 to 5, yellow line corresponds to BF >5 to 10, green line corresponds to BF >10 to 20, and the red line corresponds to BF >20.

In 2016, WNS was detected in Washington state, over 3500 kilometers of aerial distance if we take New York state as the reference location where the disease was first reported [35]. In this study, we used the virus information from the isolate (ID:27099) first reported from Washington along with an isolate from 2017 and several isolates from 2018. The time referenced Bayesian tree showed that PdPV-pa isolates from Washington were phylogenetically close to an isolate (27WNS_KY_NLE_2012) sampled in 2012 from Breckinridge County, Kentucky (Fig 4). All of these isolates shared a common ancestor with PdPV-pa isolates collected from Mammoth Cave, Kentucky in 2018. The whole clade had posterior support of 100% (Fig 4). The result was further supported by a high BF value of 20.6 in the BSSVS analysis (Table 2) for the spread from Kentucky to Washington state. Further, Fig 5 illustrates all significant paths of spread that have BF≥3. Locations such as Connecticut, New York, Kentucky, and West Virginia that appeared as major source locations in the MCC tree were also supported in the BSSVS analysis with significant BF values and higher connectivity.

**Table 2:** Bayes factors of the location pairs with ≥ 3 values and their respective posterior probability.

From	To	Bayes factor	Posterior probability
CT^1^	VT	3.0	0.2
CT	IL	3.6	0.2
NY	IL	3.8	0.3
CT	WV	3.8	0.3
NY	PA	4.5	0.5
IL	AL	4.6	0.4
IL	MO	4.7	0.4
KY	IL	5.4	0.4
NY	NC	5.4	0.4
CT	OH	5.9	0.4
WV	IN	6.8	0.5
WV	NC	10.8	0.6
NY	GA	12.2	0.6
NY	IN	14.0	0.7
KY	WA	20.6	0.7
CT	NB	20.3	0.7
KY	OH	34.9	0.8
WV	VA	95.6	0.9
CT	KY	134.8	0.9

1 Standard abbreviations for US states are used in the table except for New Brunswick province of Canada abbreviated as NB.

### Time to the most recent common ancestor, ancestral location, and the substitution rate

Ancestral reconstruction in the discrete phylogeographic analysis with BEAST supports the average time to the most recent common ancestor (TMRCA) of PdPV-pa was 1999 with 95% HPD interval ranging from 1996 to 2002 (S3 Fig). The TMRCA estimated from TempEst was close to the 95% HPD interval. As noted above, the location-annotated MCC tree (Fig 1) showed no single location dominated across the tree. The probability distribution of the root locations (retrieved from the summary tree location file in our BEAST analysis) showed a range from 0.01 to 0.12 (Table 3). Some locations like Connecticut, Alabama, South Carolina, New York, and Illinois showed relatively higher probabilities (0.12, 0.11, 0.09, 0.08, and 0.08 respectively) than the rest. However, the probabilities were small in magnitude and the difference among locations was so small that none of the locations can be justified to call as a root location. In the analysis, the mean substitution rate for the virus was determined to be 2 X 10^−3^ substitutions per site per year with 95% HPD interval ranging from 1.5 X 10^−3^ to 2.3 X 10^−3^ substitutions per site per year (S4 Fig).

**Table 3:** The root location probability retrieved from the summary tree location file from BEAST analysis.

Location	Probability
CT^1^	0.12
AL	0.11
SC	0.09
NY	0.08
IL	0.08
VT	0.07
NC	0.06
VA	0.06
PA	0.05
TN	0.05
KY	0.05
NB	0.04
OH	0.03
WV	0.03
IN	0.03
GA	0.02
MO	0.02
WA	0.01

1 Standard abbreviations for US states are used in the table except for New Brunswick province of Canada abbreviated as NB.

## Discussion

In this study, 139 coat protein sequences of PdPV-pa isolates obtained from Pd infected bats across 17 US states and one province (New Brunswick) of Canada were analyzed using the Bayesian phylogeographic inference framework [33]. We examined the virus spread based on its sequences sampled through time and with spatial characterization. These sequences allowed us to determine the substitution rates through time and space to infer spatial characteristics of the virus spread. We hypothesized that the phylodynamics of the virus is a reasonable approximation of white-nose syndrome spread because the virus infection is common in North American isolates of Pd; the genetic diversity of the virus is explained well by the geography [21]; the virus has a persistent lifestyle; and no incidence of the virus was found in any closely related fungi [21,36]. The shallow genetic diversity of North American population of Pd produces no spatial signatures for such analysis [18–20] hence, the genetics of the persistently infecting virus can be a significant proxy to study WNS spread and epidemiology.

The large number of samples (139 isolates from diverse locations and bat species) analyzed through the time-referenced BEAST phylogeographic tool reconfirmed our earlier results [21] that the virus isolates were geographically clustered and showed no patterns of variation associated with bat species. This implies that spread largely occurs within hibernacula, and movement to new areas is occasional. The summarized MCC tree with branches annotated with locations showed none of the locations dominating across the tree topology. This implies a lack of a precise root location where the majority of the isolates were phylogenetically linked. We did not find a clear directionality of the spread emerging from a single location in the phylogeny. It is unlikely that a single root location was significant for the spread but rather that multiple locations were involved and contributed to the epidemiology of WNS. This is evident from the SPREAD maps based on the MCC tree (Fig 3). In the map, the virus presence was evident by 1999 in Connecticut, Alabama, and in South Carolina but the major seeding events to other locations started appearing by 2005, earlier than the year WNS was first reported. A widespread occurrence of cryptic infections in some bat species without symptoms [37] and the variation in tolerance and resistance in a bat species in different sites [38] suggest the possibility of the fungus presence before the outbreak of WNS was observed. Over time (2005–2018), the dominating source locations for the virus spread emerged from Connecticut, New York, Kentucky, and West Virginia. Since the earliest sequence data of PdPV-pa infecting Pd in our analysis was from the year 2008, the spatial information of the virus prior to that was inferred based on the substitution rate algorithm created by the BEAST model. The model predicts the presence of the virus but we do not have any empirical evidence that supports the virus association with Pd and Pd transmission via bats during that time. It is likely that Pd acquired the virus infection a few years (range of 4–10 years) prior to the first report of WNS in 2006 [1,2] because when the WNS outbreak happened, Pd had already been infected with the virus. A successful microbial invasion and parasitism involve demanding events that are function of time. In general, a microbe must be able to reach the tissue it will parasitize by penetrating host tissue barriers, it should adapt and grow in the host environment, it must be able to absorb and digest components of host tissues, and it must withstand the host immune system [10,39]. Hence, although Pd appears to be well adapted to bats, given the extent of colonization of bats in other parts of the world [20] the lag of a few years before the WNS outbreak is not an unreasonable result.

The origin of the virus in Pd is unclear. A recent report of the virus in Pd isolates from the Czech Republic [36] may suggest an alternative explanation to its origin in North America: that the virus came with European introduction of Pd. Previously we screened eight of the 18 Czech Republic isolates tested in the report and we did not find the virus in any of the isolates [21], but this report found five positive out of the eight. Unfortunately no genetic analysis of the fungus was conducted in this report, which would have helped to ascertain that these isolates were truly unique. The likelihood of finding the exact isolate that initiated the Pd infection in North America is extremely remote. Moreover, the report [36] did not describe any genetic variation in the virus from the Czech Republic that had phylogenetic signatures suggesting an ancestral relationship with the US isolates. Testing for the virus in a statistically valid pool from Europe is needed, as other European isolates did not have the virus.

Our analyses indicate that the virus-infected Pd established in local populations and maintained its lineages with mutations. The locations (here US states) with higher connectivity such as Connecticut, New York, Kentucky, and West Virginia appeared as major sources for the WNS spread. This dynamic can be described as a metapopulation model of patchy spread rather than a diffusive dispersal. It further suggests that the history of spread in each location is independent. This explains why the virus isolates from Tennessee appear as a separate clade from the rest. The virus isolates from Tennessee were similar to the root as suggested by a strong posterior support in MCC tree but lacked support for being the origin due to the low probability value for the root state. Diverse patterns of spatial spread have been identified depending on the ecology of pathogens and hosts. For example, a stratified diffusion pattern was evident in rabies virus [40], a random jump was found in the foot-and-mouth disease virus [41] and a diffusive spread was reported in the West Nile virus [42]. In addition, a combination of local diffusion and infrequent long-distance spread was shown in the sudden oak death pathogen, *Phytopthora ramorum* [43]. Maher et al. [11] applied maximum likelihood estimates on the county scale infection data of WNS in the United States and came to a similar conclusion that WNS spread is not diffusive but patchy. The authors found that the geographic heterogeneity associated with cave connectivity and winter length were the best predictors for their model. A multiscale process involving stochasticity in the spread of WNS was also emphasized in a recent study focusing on the data from 54 hibernacula of little brown bats in New York [14]. The patchy spread fits well with the stochasticity associated with multiscale processes because of the lack of deterministic directionality found in the phylogeny from a single location. Our analysis identifies the source locations for seeding and illustrates the temporal dynamics of the patchy spread that were not captured by previous studies.

In order to verify that our results were not biased by our sampling intensity, we created a random sample subset excluding the states with single isolates and selected only states that had at least two samples collected in different times. We ran the subset for BEAST phylogeographic and SPREAD analysis. Our results showed similar phylogenetic support and topological relationships among the virus isolates as we found with full data set (S5 Fig) and the patchy spread pattern of the virus with major seeding locations from Connecticut, New York, West Virginia and Kentucky was evident (S6 Fig). The SPREAD map also inferred presence of the virus in 1999 both in Alabama and New York states (S7 Fig). The model with full data set did not predict the virus in New York state in 1999 but the model with the new subset inferred this because the samples from Connecticut and South Carolina were dropped and the inferred substitutions for New York and Alabama samples came closer in genetic distance. The inference of the virus in different locations before WNS was first reported from New York may suggest multiple introduction of Pd. However, our current analysis could not provide enough evidence for that. The model predicts the presence of the virus prior 2006, but lacks clear evidences for its source.

The recent long-distance spread of WNS to Washington state appeared in 2016 from the field data. Our phylogeographic approach in BEAST reasonably traced the source of the spread to Kentucky that had close sequence similarity with the virus isolates from Mammoth Cave. The long distance spread of the virus is likely mediated by humans because Mammoth Cave National Park is one of the most heavily visited national parks in the United States [44]. The fungal mycelia or conidia could be transmitted through clothing, footwear or various gear used by cavers.

Although the geographical origin of the virus was not definite, we performed BSSVS analysis that provided BF tests to evaluate epidemiologically significant non-zero migration rates between locations. The epidemiological linkages that correspond to BF≥3 identified Connecticut, Illinois, New York, Kentucky, and West Virginia as significant source states for the spread. Some transmissions such as Kentucky to Washington, Connecticut to New Brunswick, Kentucky to Ohio, West Virginia to Virginia, and Connecticut to Kentucky showed very high BF values of over 20, indicating that those spreads were extremely likely. Moreover, states like Connecticut, New York, West Virginia, and Kentucky identified in the MCC tree as the dominating seeding location for the virus movement were also supported by BSSVS analysis.

The time-referenced discrete phylogeographic analysis in BEAST also provided some important evolutionary information about PdPV-pa. We inferred the TMRCA of the virus was somewhere between the late 1990s to early 2000s (Mean = 1999, 95% HPD interval [1996, 2002]) in North America. This suggests that the virus was present and existed before WNS spread happened. The lag period for WNS emergence likely coincided with the period of the virus infection of the fungus. The average nucleotide substitution rates as determined in BEAST analysis for the virus’s coat protein gene (Mean: 2 X 10^−3^ substitutions per site per year, 95% HPD interval [1.5 X 10^−3^, 2.3 X 10^−3^]) is within the order of magnitude for dsRNA viruses (~10^−7^ to 10^−3^) reported earlier [45]. Although the substitution rate for dsRNA viruses, particularly for mycoviruses, is less known, the PdPV-pa substitution rate in the order of 10^−3^ is very high for a virus with a persistent lifestyle and a stamping-machine mode of replication [46–48]. The higher substitution rate might reflect the recent infection history of the virus in Pd that seems likely from the recent origin of the virus in North America. Most viruses exhibit a higher level of variation after recent species jumping [49,50]. Our data does not allow us to determine a definitive origin for the virus, but it does imply that the virus only recently infected Pd. This could mean that the fungus arrived in North American without the virus, and acquired it from a related native North American fungus. Given the impact of the virus on sporulation [21], this could have led to the spread of WNS from the original site of Pd infection. Unfortunately finding the progenitor of the virus is a nearly impossible task.

The spatial and temporal characteristics of WNS spread described based on the sequence information of PdPV-pa have important management implications. Our analysis depicts the spread of WNS driven by the dynamics of some patchy locations over time. It is important to identify such locations to implement rigorous measures to control Pd and the subsequent spread of the disease. In our current analysis over 10 years, Connecticut, New York, West Virginia, and Kentucky were identified as major patches with higher connectivity both in the MCC tree and BSSVS analysis. Some of the locations such as Kentucky seem more relevant for management purposes as it appears as a major source for the long-distance dispersal of the fungus to Washington. We identify Mammoth Cave at a local scale as a significant location for possible human-mediated dispersal. We also noted Illinois as a significant location for a non-zero migration rate in BSSVS analysis but, unlike other locations, it is not supported in the MCC tree. A regular update of the time-structured phylogeographic analysis as new data become available would be a reliable tool for prioritizing management interventions in controlling WNS.

In conclusion, this study outlines a framework of how a persistent virus infecting a pathogenic fungus could be a helpful tool to understand the epidemiology of the fungal disease. We have shown the importance of the time-referenced phylogeographic approach in BEAST to evaluate the temporal and spatial dynamics of WNS spread and to the management of the disease. The approach will be equally relevant to examine the efficacies of competing mitigation strategies imposed in controlling the disease.

## Supporting information

S1 Fig The root-to-tip regression using molecular divergence data of Pseudogymnoascus destructans partitivirus-pa isolates and sampling dates in TempEst. The positive slope suggests a positive correlation between the divergence and sampling dates. The X-intercept value corresponds to the time to the most recent common ancestor (TMRCA).
(PPTX)

S2 Fig The graphical outcome from bModelTest in BEAST showing support of Pseudogymnoascus destructans partitivirus-pa sequences to 30 reversible substitution models with symmetric rate tested. The models with blue circles are inside 95% HPD, red outside, and without circles have at most 0.89% support. Note model:121321 that is close to the HKY model has the highest posterior support. The posterior and cumulative support values of the major models are listed in a side table.
(PPTX)

S3 Fig Frequencies of maximum clade credibility (MCC) tree-heights plotted as an output from Tracer v1.7. The statistics from the analysis are summarized in an inset. Given the mean tree-height of almost 19 years and 2018 is the collection year of our youngest sample, the average time to the most recent common ancestor (TMRCA) is calculated as 1999 with 95% HPD [1996, 2002].
(PPTX)

S4 Fig Frequencies of clock-rate as evaluated in Tracer v1.7 from the data retrieved from the phylogeographic analysis in BEAST. The statistics from the analysis are summarized in an inset which showed the mean substitution rate for *Pseudogymnoascus destructans* partitivirus-pa as 2X10^-3^ substitution per site per year with 95% HPD [1.5X10-3, 2.3X10-3].
(PPTX)

S5 Fig The strict clock phylogeographic tree constructed from a subset (randomized 134 samples) of coat protein sequence of Pseudogymnoascus destructans partitivirus-pa (PdPV-pa) isolates in BEAST v2.5. The tree is annotated with colors coded for the locations that included 12 US states and New Brunswick, Canada. Standard abbreviations of US states are used in the figure legend except “Can” for New Brunswick province of Canada and “KYmc” for Mammoth cave locations in Kentucky. Nodes of the tree are denoted by solid black circles and the size of the circle corresponds to posterior supports shown in the legend. Branch lengths are in time, depicted by a horizontal time-scale. For detail description of the virus isolate ID, refer to Table 1.
(PPTX)

S6 Fig The spread of white-nose syndrome in North America as predicted from Pseudogymnoascus destructans partitivirus-pa phylogeographic analysis in BEAST using a subset of 134 samples, visualized in Google Earth with SPREAD v1.0.7 and transferred manually to a USGS map. The lines connecting different locations represent branches in the maximum clade credibility tree. The bright to dark gradient of the line’s red color represent time from 1999 to 2018. Note higher number of connectivity in New York, West Virginia and Kentucky.
(PPTX)

S7 Fig A snapshot of white-nose syndrome spread in 1999 inferred from Pseudogmnoascus destructans partitivirus-pa subset sample of 134 plotted in Google Earth using SPREAD v1.0.7 and transferred to a USFS map. The line between the locations represent branches in the maximum clade credibility (MCC) tree. The dark to light color gradient of the circles indicate the relative age of the branch inferred (old to recent).
(PPTX)
