# Supplementary figures and images for: Phylogeographic analysis of Pseudogymnoascus destructans partitivirus-pa explains the spread dynamics of white-nose syndrome in North America
Source: PLoS Pathog. 2021 Mar 17;17(3):e1009236. doi: 10.1371/journal.ppat.1009236 (PMC7968715; doi:10.1371/journal.ppat.1009236)

## Slide 1
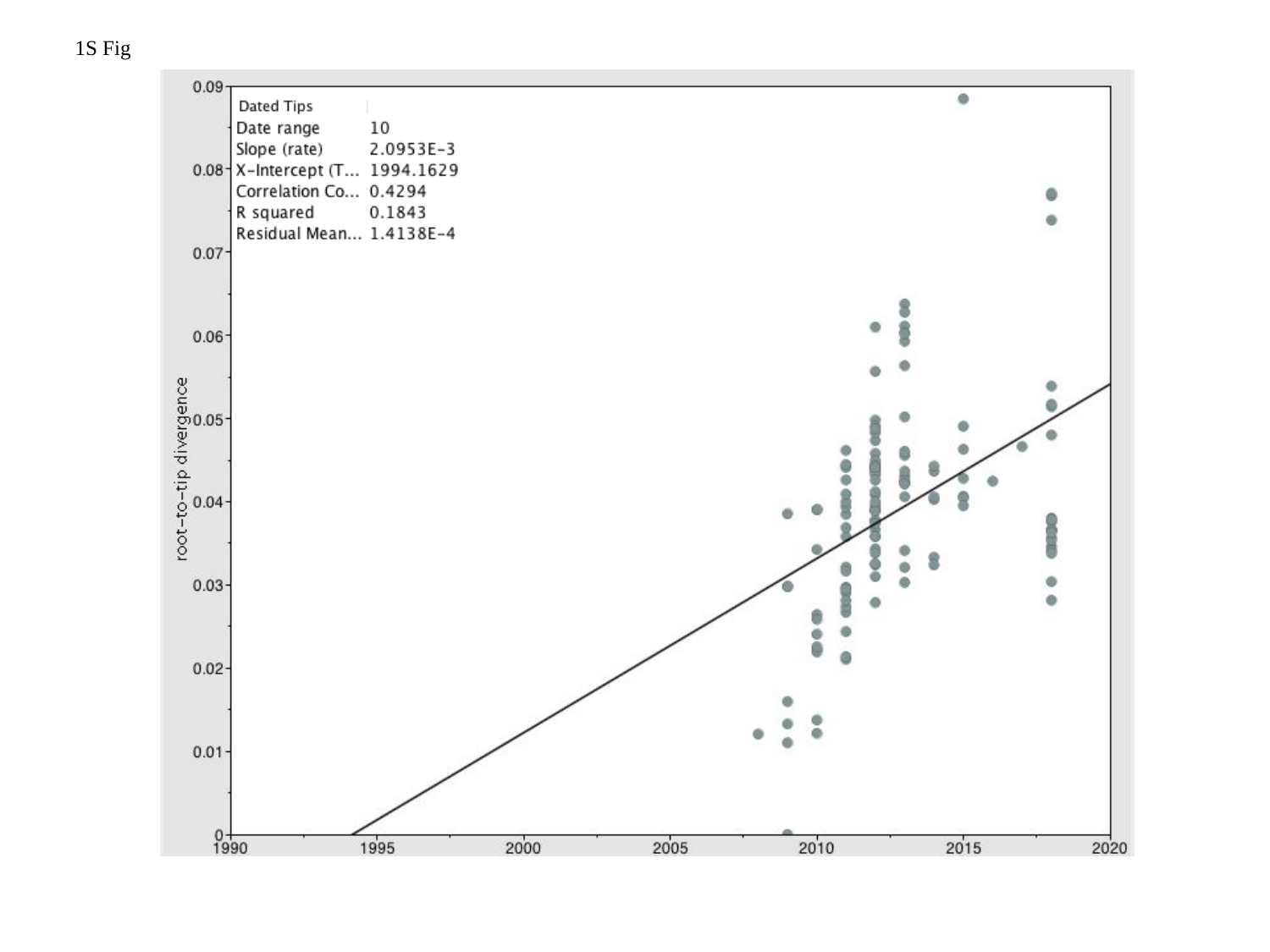

1S Fig

Supplement: S1 Fig — The positive slope suggests a positive correlation between the divergence and sampling dates. The X-intercept value corresponds to the time to the most recent common ancestor (TMRCA). (PPTX) [file ppat.1009236.s001.pptx]

## Slide 1
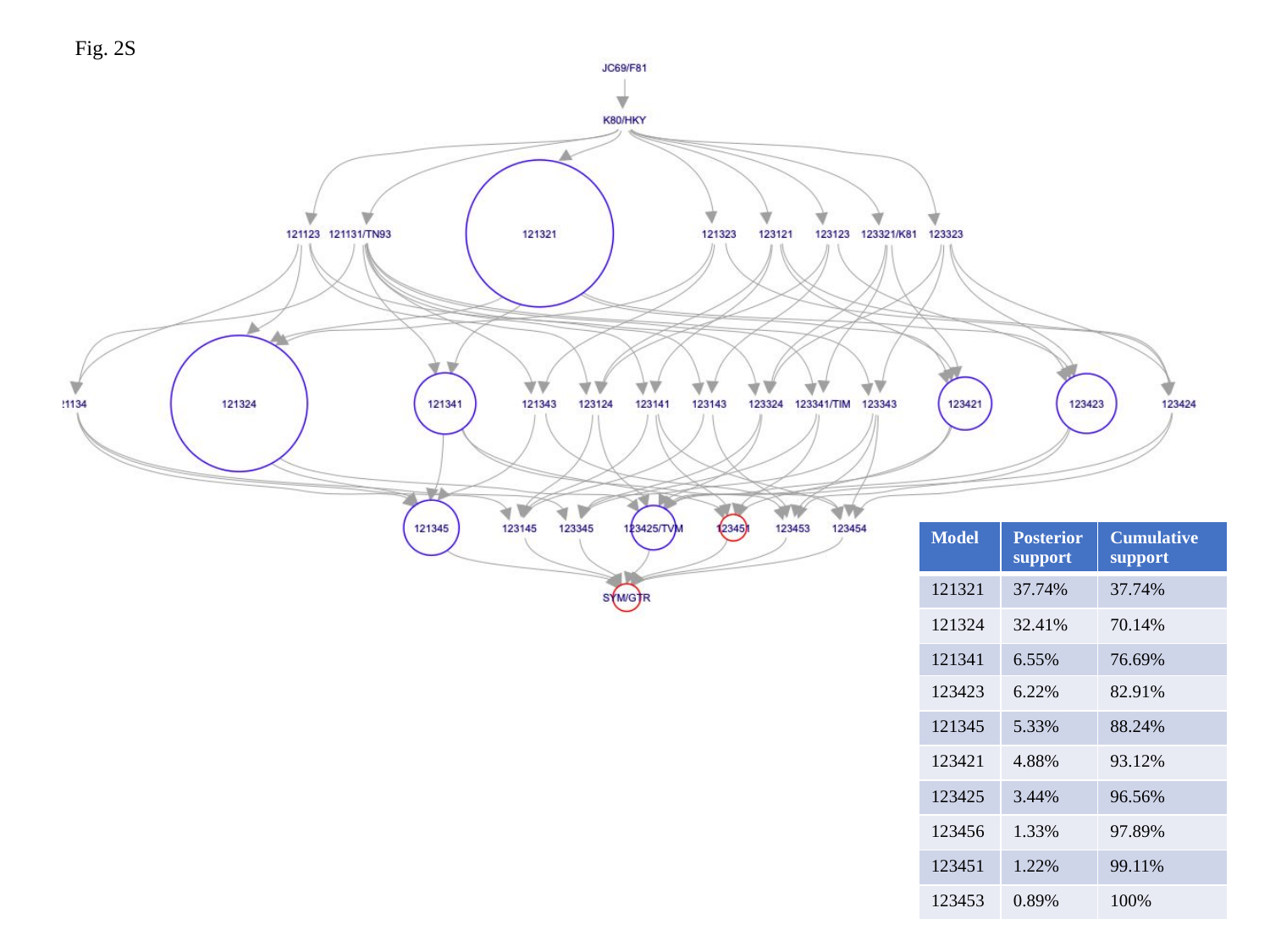

Fig. 2S
| Model | Posterior support | Cumulative support |
| --- | --- | --- |
| 121321 | 37.74% | 37.74% |
| 121324 | 32.41% | 70.14% |
| 121341 | 6.55% | 76.69% |
| 123423 | 6.22% | 82.91% |
| 121345 | 5.33% | 88.24% |
| 123421 | 4.88% | 93.12% |
| 123425 | 3.44% | 96.56% |
| 123456 | 1.33% | 97.89% |
| 123451 | 1.22% | 99.11% |
| 123453 | 0.89% | 100% |

Supplement: S2 Fig — The models with blue circles are inside 95% HPD, red outside, and without circles have at most 0.89% support. Note model:121321 that is close to the HKY model has the highest posterior support. The posterior and cumulative support values of the major models are listed in a side table. (PPTX) [file ppat.1009236.s002.pptx]

## Slide 1
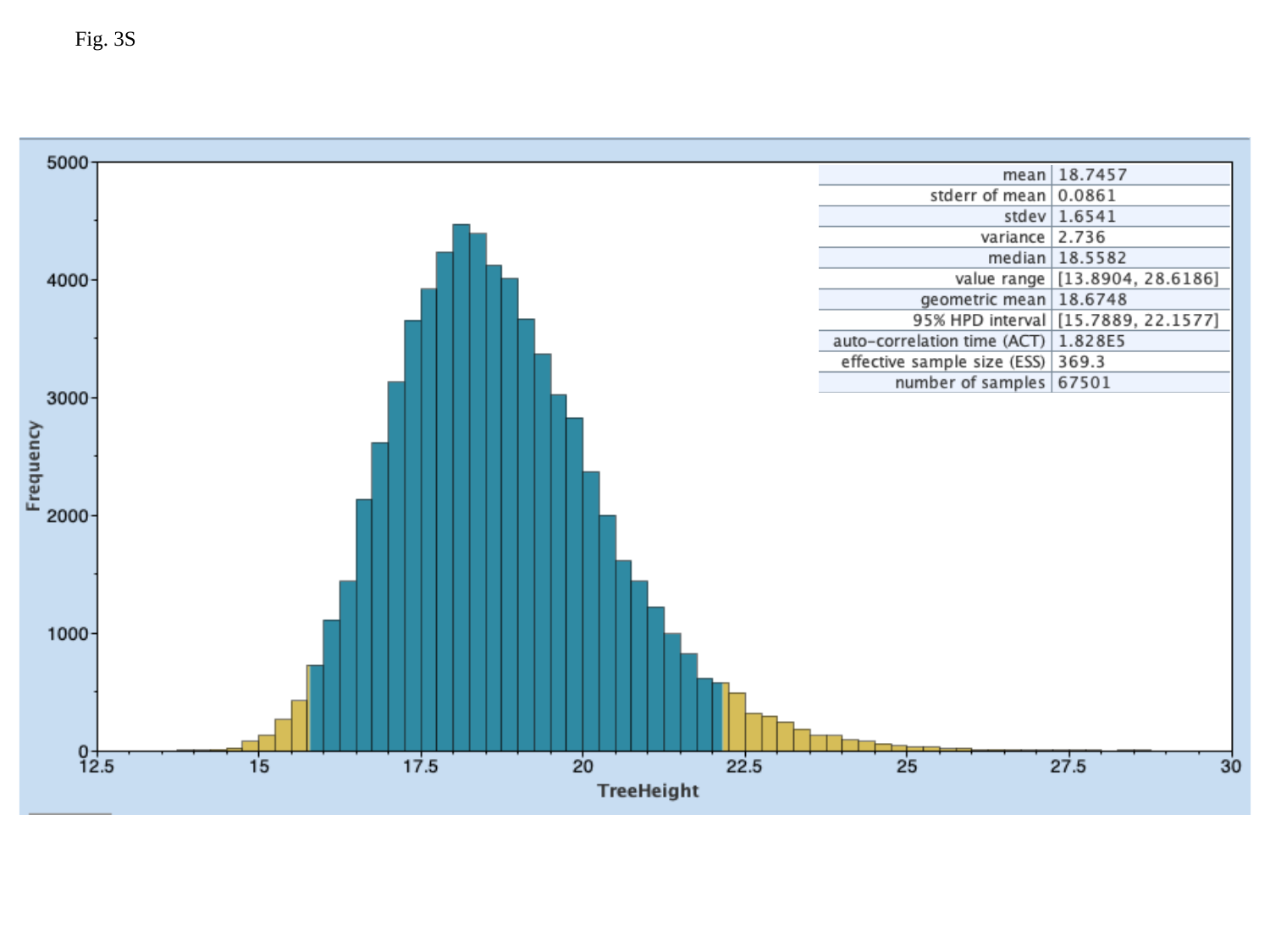

Fig. 3S

Supplement: S3 Fig — The statistics from the analysis are summarized in an inset. Given the mean tree-height of almost 19 years and 2018 is the collection year of our youngest sample, the average time to the most recent common ancestor (TMRCA) is calculated as 1999 with 95% HPD [1996, 2002]. (PPTX) [file ppat.1009236.s003.pptx]

## Slide 1
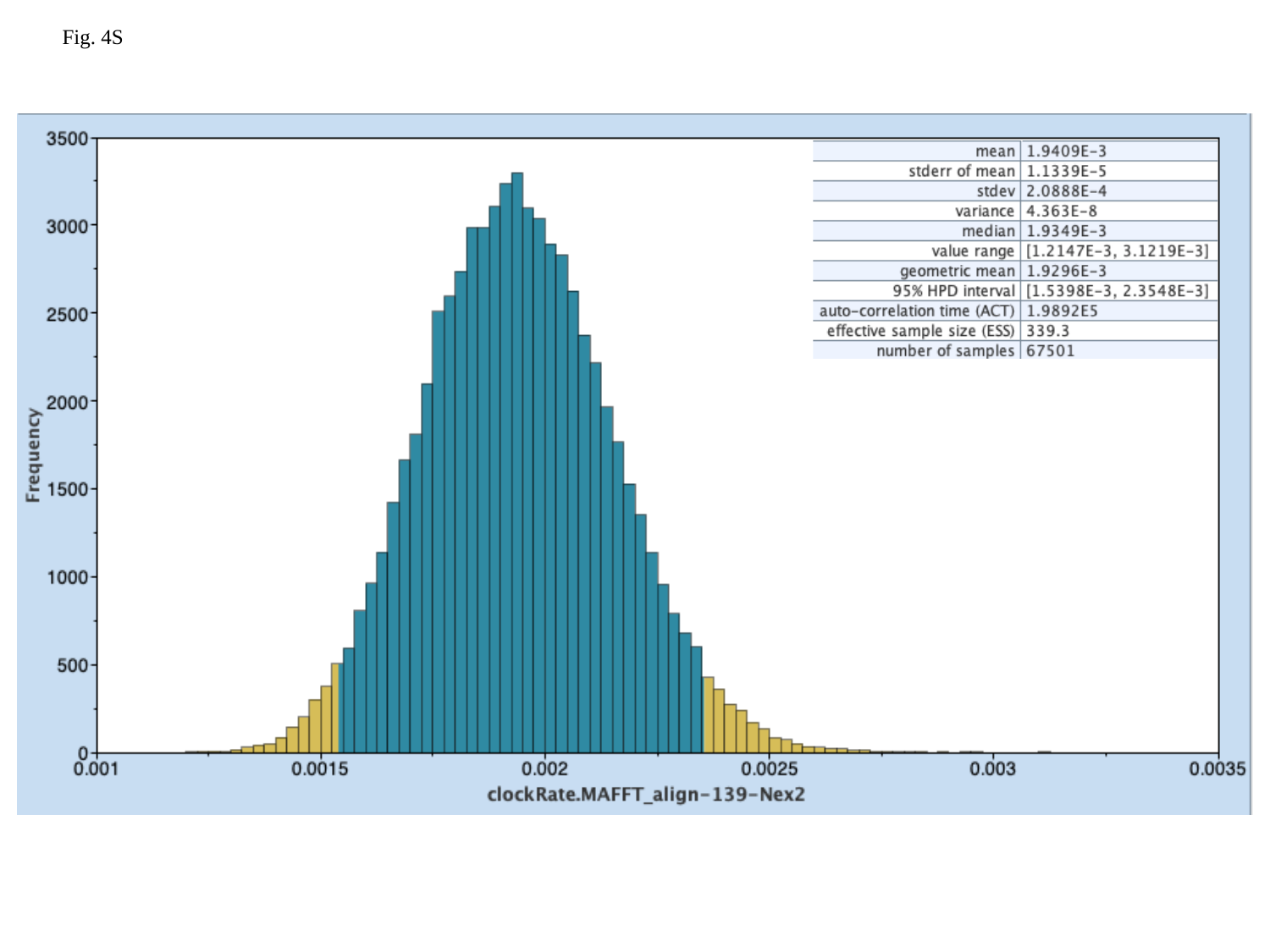

Fig. 4S

Supplement: S4 Fig — The statistics from the analysis are summarized in an inset which showed the mean substitution rate for Pseudogymnoascus destructans partitivirus-pa as 2X10-3 substitution per site per year with 95% HPD [1.5X10-3, 2.3X10-3]. (PPTX) [file ppat.1009236.s004.pptx]

## Slide 1
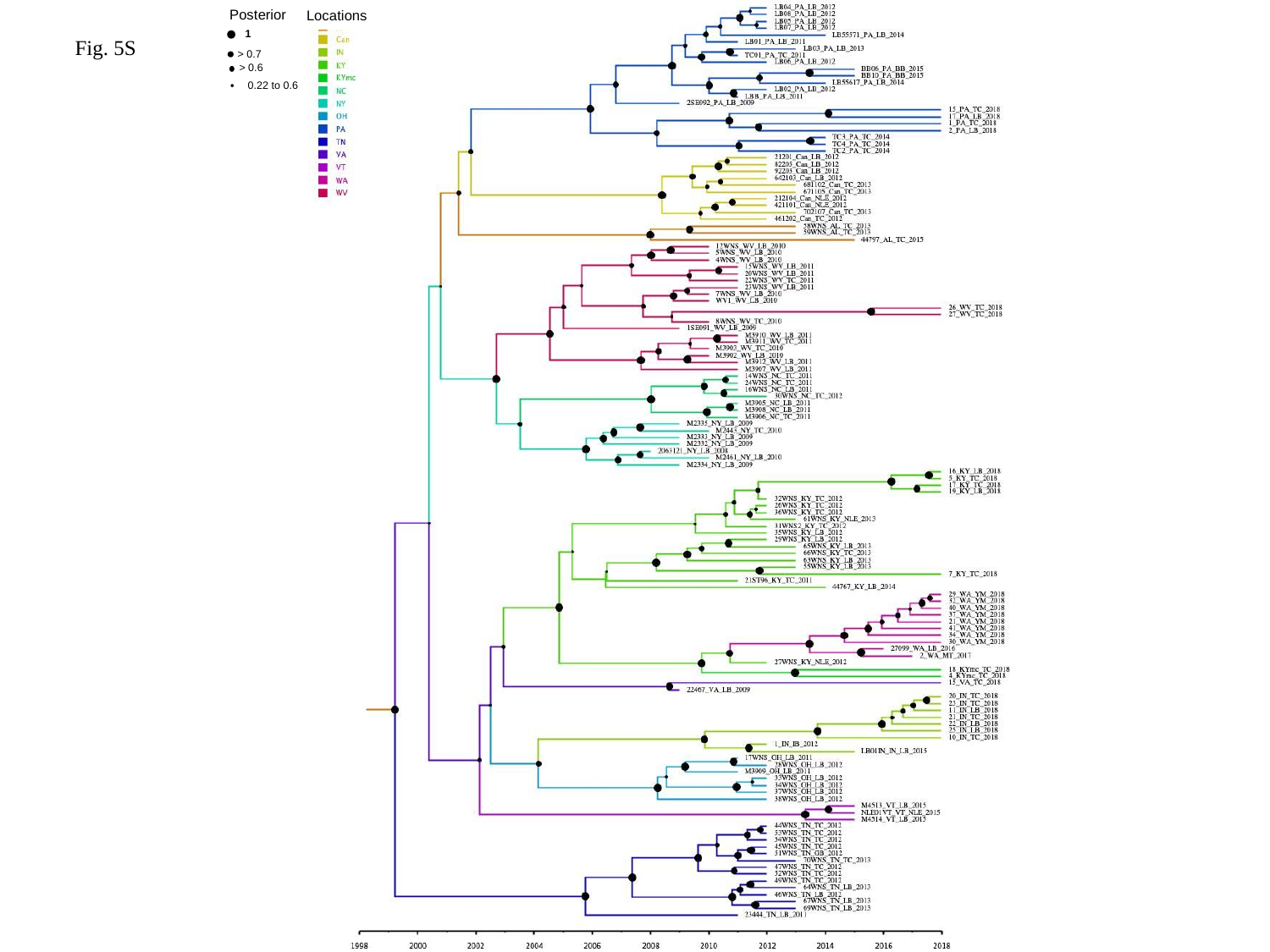

Posterior
1
> 0.7
> 0.6
0.22 to 0.6
Locations
Fig. 5S

Supplement: S5 Fig — The tree is annotated with colors coded for the locations that included 12 US states and New Brunswick, Canada. Standard abbreviations of US states are used in the figure legend except “Can” for New Brunswick province of Canada and “KYmc” for Mammoth cave locations in Kentucky. Nodes of the tree are denoted by solid black circles and the size of the circle corresponds to posterior supports shown in the legend. Branch lengths are in time, depicted by a horizontal time-scale. For detail description of the virus isolate ID, refer to Table 1. (PPTX) [file ppat.1009236.s005.pptx]

## Slide 1
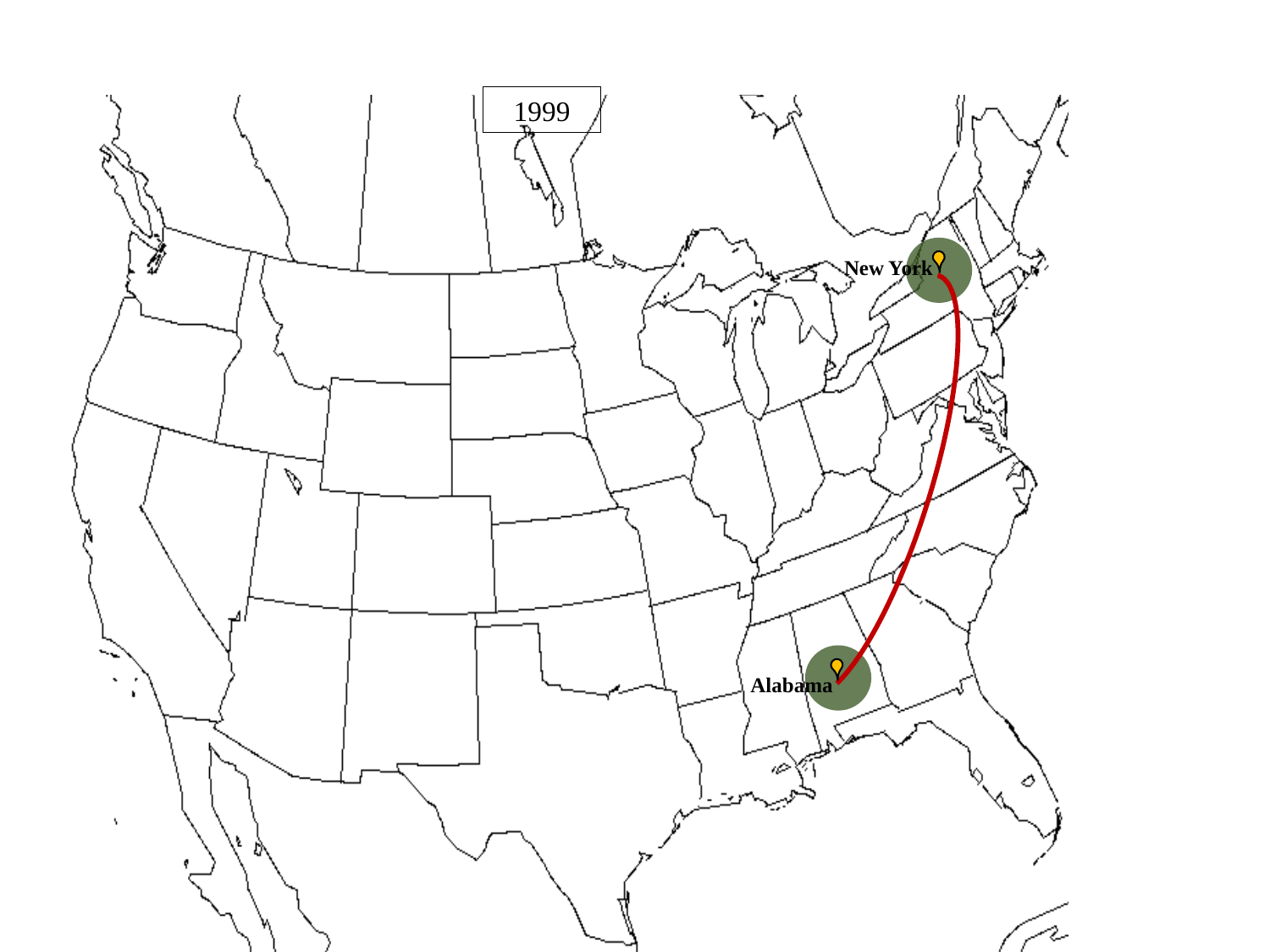

1999
New York
Alabama

Supplement: S7 Fig — The line between the locations represent branches in the maximum clade credibility (MCC) tree. The dark to light color gradient of the circles indicate the relative age of the branch inferred (old to recent). (PPTX) [file ppat.1009236.s007.pptx]
