# Supplementary material for: Phylogeographic analysis of Pseudogymnoascus destructans partitivirus-pa explains the spread dynamics of white-nose syndrome in North America
Source: PLoS Pathog. 2021 Mar 17;17(3):e1009236. doi: 10.1371/journal.ppat.1009236 (PMC7968715; doi:10.1371/journal.ppat.1009236)
Supplement: S6 Fig — The lines connecting different locations represent branches in the maximum clade credibility tree. The bright to dark gradient of the line’s red color represent time from 1999 to 2018. Note higher number of connectivity in New York, West Virginia and Kentucky. (PPTX) [file ppat.1009236.s006.pptx]

## Slide 1
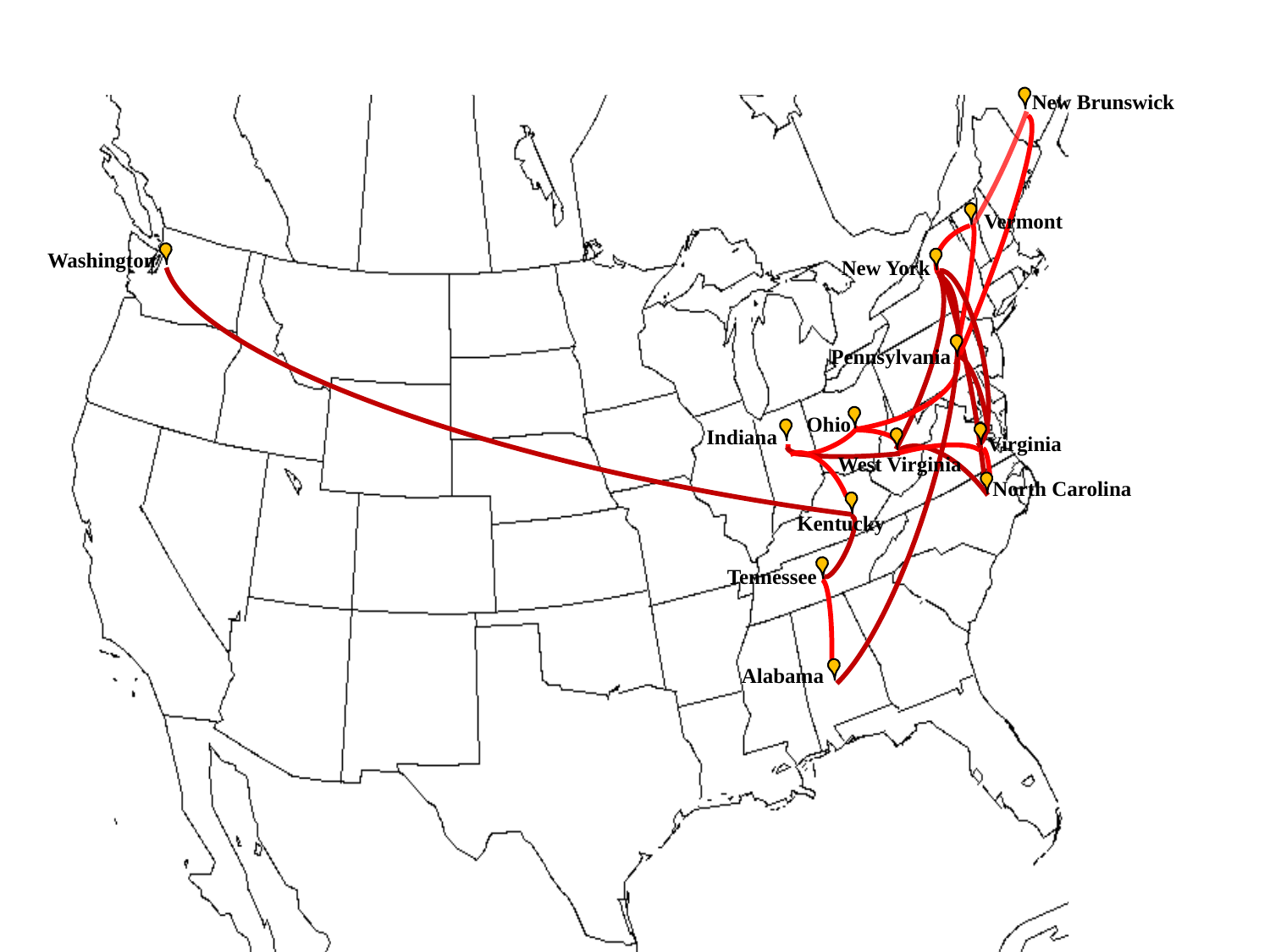

New Brunswick
Vermont
Washington
New York
Pennsylvania
Ohio
Indiana
Virginia
West Virginia
North Carolina
Kentucky
Tennessee
Alabama
